# Predicting health-related quality of life in cancer patients receiving chemotherapy: a structural equation approach using the self-control model

**DOI:** 10.1186/s12913-017-2675-4

**Published:** 2017-11-09

**Authors:** Yu-Ri Park, Eun-Young Park, Jung-Hee Kim

**Affiliations:** 1 Dankook University Hospital, 201 Manghyang-ro, Dongnam-gu, Cheonan, Chung Nam 31116 South Korea; 2 Department of Secondary Special Education, College of Education, Jeonju University, 45 Baengma-gil, Wansan-gu, Jeonju, 560-759 South Korea; 3 College of Nursing, The Catholic University of Korea, 222 Banpo-daero, Seocho-gu, Seoul, 06591 South Korea

**Keywords:** Cancer, Chemotherapy, Neuropathy, Self-control, Quality of life

## Abstract

**Background:**

According to the self-control model, self-control works as a protective factor and a psychological resource. Although an understanding of the effect(s) of peripheral neuropathy on quality of life is important to healthcare professionals, previous studies do not facilitate broad comprehension in this regard. The purpose of this cross-sectional study was to test the multidimensional assumptions of quality of life of patients with cancer, with focus on their self-control.

**Methods:**

A structural equation model was tested on patients with cancer at the oncology clinic of a university hospital where patients received chemotherapy. A model was tested using structural equation modeling, which allows the researcher to find the empirical evidence by testing a measurement model and a structural model. The model comprised three variables, self-control, health related quality of life, and chemotherapy-induced peripheral neuropathy. Among the variables, self-control was the endogenous and mediating variable.

**Results:**

The proposed models showed good fit indices. Self-control partially mediated chemotherapy-induced peripheral neuropathy and quality of life. It was found that the physical symptoms of peripheral neuropathy influenced health-related quality of life both indirectly and directly.

**Conclusions:**

Self-control plays a significant role in the protection and promotion of physical and mental health in various stressful situations, and thus, as a psychological resource, it plays a significant role in quality of life. Our results can be used to develop a quality of life model for patients receiving chemotherapy and as a theoretical foundation for the development of appropriate nursing interventions.

## Background

It has been reported that the 5-year relative survival rate of patients with cancer was 69.4% in 2014, with 2 out of 3 patients surviving for 5 years or more [1]. The survival rate tends to increase with advances in medical treatments, and the quality of life (QOL) during the survival period is considered as a high priority.

It is well known that the symptoms that occur after a cancer diagnosis and during treatment cause physical discomfort and affect the performance of daily activities, as well as health-related QOL [2, 3]. Particularly, peripheral neuropathy is one of the complications that patients with cancer often experience during chemotherapy [3]. Symptoms of peripheral neuropathy, which include pain, muscle weakness, muscle atrophy, urinary and fecal incontinence, erectile dysfunction, and intestinal obstruction, affects daily life [4].

A systematic review revealed that there is some discordance between the results of studies on the association between QOL and peripheral neuropathy symptoms resulting from chemotherapy [5]. A study on patients with lung cancer in Japan has reported that peripheral neuropathy has no association with functional, physical, cognition, and psychosocial areas [6], and others reported the significant association between peripheral neuropathy and physical, psychosocial, emotional, social, and academic areas [7].

Most of the studies included in the systematic review did not test the influence of chemotherapy-induced peripheral neuropathy (CIPN) symptoms on QOL directly [5]. Moreover, although pain, which is the main symptom of peripheral neuropathy, affects participation in social, leisure, and family activities, it is not clear as to how physical symptoms affects QOL-related areas such as psychological well-being [8]. In the existing literature, it is not unclear to find the causal relationship between the symptoms of CIPN and QOL.

According to the self-control model, self-control works as a protective factor, and thus, is a psychological resource. It has also been reported that the strengthening self-control positively influences individuals’ psychological wellbeing and behavior [9, 10]. A hypothetical model was developed based on Baumeister’s strength model of self-control [11] and a literature review. Self-control refers to the ability to change one’s response, including the pursuit of standards such as ideals, value, ethics, and social expectations, or long-term goals. In the self-control strengthening model, the function of self-control is likened to that of a muscle, such that energy is used sparingly when a muscle is tired, though utilization is maximized when the motive for what one wants, or is pursuing, is strengthened [11].

In other words, self-control has a buffering function, demonstrated by the protection of an individual from the negative effect(s) of resistance to stress; there is a considerably higher reduction in self-control due to disease, as opposed to that observed in a non-clinical population [10]. Self-control constitutes understanding and controlling one’s functions and instincts, and it is associated with not only human behavior but also with emotional problems, attachment, patience, failure of performing tasks, and relationships [11]. An understanding of self-control can be applied in various areas of human lives. As a cognitive factor among humans, the self-control process is a significant defense mechanism, mediating human behavior and psychological changes, with the potential to affect health and through the promotion of happiness and success [1].

Although the cancer patient experiences the pain, which is the main symptom of peripheral neuropathy and result in loss of inhibitory capacity, the patient with more sense of control could not lose their capacity to focus and concentrate. Based on this buffering effect of self-control, this variable was selected as a mediator of the association between physical symptoms and QOL in the present study.

However, a study on the association between peripheral neuropathy and a multi-dimensional concept such as QOL would yield results with possible limitations in terms of the assessment, interpretation, and application. Therefore, a model was tested using structural equation modeling (SEM), which allows the researcher to find the empirical evidence by testing a measurement model and a structural model. The aim of the present study was to test the multidimensional assumptions of QOL in patients with cancer with focus on self-control.

This study examined the role of degree of peripheral neuropathy and self-control as variables affecting QOL, through a theoretical hypothesis and a literature review, and developed a hypothetical model using these variables. Moreover, the study sought to clarify the QOL of peripheral neuropathy patients through verification of the partial and full mediating effects of self-control, using the self-control strengthening model.

## Methods

### Participants

This study used a cross-sectional design with a convenience sample comprising patients with cancer. The following were the exclusion criteria for this study: 1) receiving chemotherapy within the past 2 years and 2) not being diagnosed with peripheral neuropathy before chemotherapy. Subjects provided information indicating whether they had undergone chemotherapy and if they had a history of peripheral neuropathy; this information was verified through a review of their medical records, to which each subjects consented.

As the number of subjects is calculated as a 5:1 ratio with the number of free parament under study [12], it was determined that more than 75 patients were required in this study. Ninety-four patients consented to data collection, and one questionnaire was excluded from the analysis due to incomplete data. Thus, 93 subjects were included in this study and their general and clinical characteristics were as follows <Table 1>. With regard to subjects’ sex, there were 53 (57.0%) males and 40 (43.0%) females. The mean age was 59.35 years, and the age distribution indicated that the largest age group was 50–59 years, which comprised 27 subjects (29.0%). Sixty-eight (73.1%) patients were receiving chemotherapy at the time of data collection. The mean number of chemotherapy sessions was 9.5, with ≤5 sessions per cycle being the most common, which was reported by 43 subjects (46.2%). Dose reduction during chemotherapy occurred for 30 subjects (32.2%); there was no dose reduction for 63 of the subjects (67.7%).

**Table 1:** General and clinical characteristics of subjects

Characteristics	Categories	n (%)	Mean ± SD
Gender	Male	53(57.0)	
	Female	40(43.0)	
Age (yrs)	<40	6(6.5)	59.35 ± 12.27
	40–49	12(12.9)	
	50–59	27(29.0)	
	60–69	25(26.9)	
	≥70	23(24.7)	
Smoking	Yes	11(11.8)	
	No	82(88.2)	
Alcohol	Yes	4(4.3)	
	No	89(95.7)	
Diagnosis type of cancer	Gastric cancer	9(9.7)	
	Colon cancer	9(9.7)	
	Head or neck cancer	13(14.0)	
	Gynecologic cancer	9(9.7)	
	Esophageal cancer	13(14.0)	
	Lung cancer	5(5.4)	
	Hematologic cancer	25(26.9)	
	Others	10(10.8)	
Status of chemotherapy	Current	68(73.1)	
	Past	25(26.9)	
Cycles of chemotherapy	≤5	43(46.2)	9.58 ± 11.32
	6–10	22(23.7)	
	11–15	14(15.1)	
	16–20	6(6.5)	
	≥21	8(8.6)	
Chemoagent dose reduction	Yes	29(31.2)	
	No	63(67.7)	

### Data collection

Participants were selected based on the study’s inclusion criteria, among out-patients and in-patients who had previously received or were receiving chemotherapy. The data were collected during 2013. Subjects completed the self-administered questionnaires and either a researcher or research assistant read questionnaires or provided additional explanations, if needed.

### Measures

The Korean version of the Quality of Life Questionnaire Chemotherapy-Induced Peripheral Neuropathy (QLQ-CIPN20) [13] was used to assess the degree of CPIN and functional limitations experienced by the subjects [14]. This tool determines the degree of subjects’ peripheral neuropathy through 20 questions comprising 3 subcategories; the sensory, motor, and autonomic subscales. Each item is rated on a 4-point scale and is converted to the 0–100 scale. A higher score indicates more severe symptoms and discomfort. The Cronbach’s alpha for this scale were .74~85 in this study.

Self-control was measured using the Mastery Scale [15]. The scale contains 7 items on which participants indicate agreement or disagreement using a 4-point scale. “I have little control over the things that happen to me” and “I can do just about anything I really set my mind to do” are some of the scale’s items. Five items were reverse scored and the total score ranged from 7 to 28 points. Higher scores indicated stronger feelings of mastery. A Cronbach’s alpha value of .82 indicated good reliability in this study.

The health related quality of life was measured by the Korean version of the QLQ-C30 (3.0 version) [16], which were originally developed by the European Organization for Research and Treatment of Cancer (EO-RTC) [17]. This multitrait scale consists of 3 scales, namely, global QOL, functioning, and symptom scales. It consists of 30 questions, each answered on a 4-point scale, and those relating to global QOL are rated on a 7-point scale. Following conversion to the 0–100 score range, according to the tool’s rating guidelines, higher scores on global QOL and the functional domain, and a lower score on symptoms indicate higher QOL. The Cronbach’s alpha for this scale was .72~89 in this study.

### Ethical considerations

The study was conducted upon approval by the Institutional Review Board of D university hospital in C city.

### Data analysis

Structural equation modeling was used to test a measurement model and a structural model. Data were analyzed using the AMOS (Chicago, IL, USA) version 22 software package to obtain maximum-likelihood estimates of model parameters and goodness-of-fit indices.

The model comprised three variables, self-control, QOL, and CIPN. Among the variables, self-control was the endogenous and mediating variable. The structural equation modeling was divided into two parts, the structural and measurement model. The measurement model was employed to verify the relations between the latent or unobserved variables and their measures [18]. The structural model was employed to verify the relationships between variables. The maximum-likelihood estimation method was employed to examine the model fit.

### Validity and reliability

This study was conducted using instruments that had been found valid and reliable for use with Korean patients with cancer. Additionally, an examination of the measurement model showed an adequate relationship between latent variables and their measures.

## Results

### Correlation coefficients

The correlation matrix for all the variables has been shown in Table 2. The correlation coefficients were identified as significant between six variables (*p* < 0.01), except between the sensory domain in CIPN and general global QOL, and the significant correlations exhibited a relationship in the expected directions. There was no multi-collinearity, which means that bivariate correlations exceeded 0.90.

**Table 2:** Inter-correlations of variables

Variables	Motor	Autonomic	Self- control	Global QOL	Functioning	Symptom
Sensory	.447^**^	.737^**^	−.467^**^	−.200	−.459^**^	−.428^**^
Motor	1	.514^**^	−.525^**^	−.359^**^	−.479^**^	−.544^**^
Autonomic	.514^**^	1	−.522^**^	−.381^**^	−.599^**^	−.542^**^
Self- control	−.525^**^	−.522^**^	1	.420**	.695^**^	.630^**^
Global QOL	−.359^**^	−.381^**^	.420^**^	1	.572^**^	.566^**^
Functioning	−.479^**^	−.599^**^	.695^**^	.572^**^	1	.863^**^
Symptom	−.544^**^	−.542^**^	.630^**^	.566^**^	.863^**^	1

Note. *QoL* quality of life

^**^ *p* < 0.01

### Testing the measurement model

Two latent variables, QOL and CIPN, were included in the measurement model. Both CIPN and QOL comprised three observed variables. A confirmatory factor analysis was employed for testing the model.

The measurement model, which tested how well the indicators of the latent variables operationalized the construct adequately, demonstrated that the tools’ ability to measure CIPN, self-control, and QOL was acceptable. The fit indices, including the normed fit index (NFI) and comparative fit index (CFI) was above 0.95 (NFI was 0.952 and CFI was 0.975). NFI and CFI were used to verify how much better the model fits than a baseline model. The CFI is independent of sample size and quantifies the amount of variation and covariation accounted for by the hypothesized model by comparing its fit to the fit of an independent model of uncorrelated variables. The root mean square error of approximation (RMSEA) was 0.102. RMSEA were used to evaluate how well a model fits the data without comparison to a baseline model [18].

Although the model fit was judged based on the fit index based on existing cut-off guidelines, these cut-offs could lead to a wrong judgment about the model [19]. Specifically, one of most common indicators, the χ^2^ test, and the RMSEA are greatly affected by sample size. If the sample size of the study is below 250, the use of RMSEA is not recommended [20]. Therefore, it is recommended to base the judgment of model fit on several indices [21]. Although the RMSEA value of above 0.10 for the measurement model was not appropriate, we accepted the hypothesis that the latent variables QOL and CIPN were measured by the observed variables because the NFI and CFI were above 0.95.

### Structural model

Incremental fit measures, including the NFI and CFI, determine how well a model fits compared with a baseline model. The estimates of model fit were χ^2^ = 21.606 (*p* = 0.042), RMSEA = 0.093, NFI = 0.946, and CFI = 0.974.

**Fig. 1:**
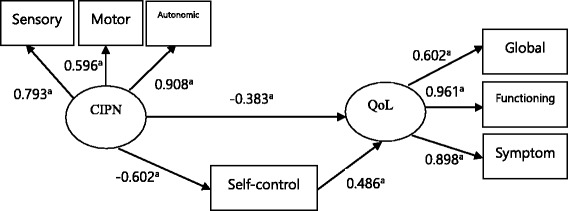
A proposed SEM model (^a^ *p* < 0.001)

The proposed SEM for this study has been shown in Fig. 1. The pathways from CIPN to self-control and QOL, and those from self-control to QOL were significant (*p* < 0.001). Table 3 summarizes the results of estimates of regression weights of the proposed model, and the direct and indirect effect of variables has been presented in Table 4.

**Table 3:** Estimates of regression weights of the proposed model

Path	B^a^	β^b^	S.E.^c^	C.R.	*p*-value
CIPN → Self-control	−.167	−.602	0.29	−5.777	<.001
Self-control → QOL	1.267	.486	.304	4.164	<.001
CIPN → QOL	−.276	−.383	.805	−3.260	.001
Global QOL → QOL	1.000				
Functioning → QOL	1.454		.216	6.733	<.001
Symptom → QOL	.471		.072	6.733	<.001
Sensory → CIPN	1.000				
Motor → CIPN	.934		.163	5.721	<.001
Autonomic → CIPN	1.144		.138	8.314	<.001

Note. *CIPN* chemotherapy-induced peripheral neuropathy, *QoL* quality of life

^a^unstandardized coefficients

^b^standardardized coefficients

^c^standard error

**Table 4:** Direct and indirect effects of variables

Predictor variables	Independent variables	Total effect	Direct effect	Indirect effect	R^2^
CIPN	Self-control	−.602^*^	.000	.000	.393
Self-control	QOL	.486^*^	.486^*^	.000	.608
CIPN		−.676^*^	−.393^*^	−.293^*^	

Note. *CIPN* chemotherapy-induced peripheral neuropathy, *QoL* quality of life

^*^ *p* < .05

Self-control and CIPN accounted for 60.8% of the variance in QOL, and CIPN accounted for 39.3% of the variance in self-control. The standardized direct effect of CIPN on QOL was −.393 and the standardized direct effect of self-control on QOL was .486. The standardized indirect effect of CIPN on QOL was −.293.

## Discussion

This is the first study testing the multidimensional assumptions of quality of life of patients with cancer with a focus on their self-control. To show potential causal relationships between latent variables, we tested the relationships between latent variables and examined a structural equation model.

The present study determined the causal relationship between the degree of CIPN, sense of control, and the quality of life of patients with cancer receiving chemotherapy. Self-control partially mediated CIPN and quality of life. In other words, it was found that the discomforting symptoms of peripheral neuropathy influenced health-related QOL both indirectly and directly. A relatively strong indirect effect of CIPN on QOL, as mediated by self-control, was found.

Based on this buffering effect of self-control, the variable was selected as a mediator of the association between physical symptoms and QOL. It is necessary to verify its role as a significant psychological resource in QOL, in addition to the effect of peripheral neuropathy, which often develops during and after chemotherapy. These results could draw the interest of oncology nurses on the role of the self-control.

Self-control is perceived control of one’s functions and behavior as central to one’s functioning, and plays a pivotal role in success in life [11]. Existing literature has shown that self-control enables the overcoming of pathological symptoms or problems in the rehabilitation of cancer survivors [22]. It also acts as a buffer and a psychological resource among patients who have limited daily activities as a result of physical disabilities [23]. A study on the mediating effect of self-control on peripheral neuropathy and the health-related QOL of lung cancer patients found that the degree of pain affects QOL through mediation by a positive psychological mechanism [24]. Self-control constitutes faith and active behavior pertaining to the notion that life can be led with one’s will and effort [25], and plays a significant role in the protection and promotion of physical and mental health in various stressful situations, and thus, as a psychological resource, it plays a significant role in QOL [26].

Peripheral neuropathy is reported in 30–40% of patients with cancer, with pain as the main symptom and it is aggravated by nutritional and metabolic imbalances, as well as by liver and renal function disorders. Particularly, anti-cancer drugs have neurotoxic effects, and, in addition to known drugs, the types of chemotherapy drugs that induce peripheral neuropathy are quite wide-ranging and vary widely [4]. Because self-control can be weakened in patients with severe symptoms and functional disabilities [11], it is necessary to enhance this mediating variable. An intervention focused on long-term symptom management that not only alleviates physical symptoms but also strengthens the patient’s psychological resources should be implemented. As there is no effective treatment for CIPN [3], cancer becomes a chronic disease [27]. A prospective study reported no time-dependent change in the QOL of patients with cancer [28]. However, the results of this study showed that an intervention aimed at strengthening self-control, a defensive mechanism mediating physical symptoms and QOL, would help improve QOL. In fact, because 30% of the pain associated with CPIN occurs in the hands and feet [29], it might be better to consider the role of self-control in the daily life of cancer patients with CIPN.

## Conclusions

This study’s investigation of the association between CPIN and QOL, with self-control as a mediating variable, will contribute to the understanding of the QOL of patients with cancer. There is a need for research on further treatment and cancer diagnosis, and therefore, on the development of a program that strengthens self-control to improve the QOL of patients receiving chemotherapy.

This cross-sectional study was conducted on patients with cancer who visit a university hospital; a possible limitation in this regard is the use of a self-administered survey. However, our model can be used to develop a QOL model for patients receiving chemotherapy and as a theoretical foundation for the development of appropriate nursing interventions.

Self-control plays a significant role in the protection and promotion of physical and mental health in various stressful situations, and thus, as a psychological resource, it plays a significant role in QOL. It is recommended that interventions focused on long-term symptom management that not only alleviates physical symptoms but also strengthens the patient’s psychological resources should be implemented.

## Abbreviations

CIPN: Chemotherapy-induced peripheral neuropathy
EO-RTC: European Organization for Research and Treatment of Cancer
GFI: Goodness-of-fit index
NFI: Normed fit index
QLQ-CIPN20: Quality of life questionnaire chemotherapy-induced peripheral neuropathy
QOL: Quality of life
RMSEA: Root mean squared error of approximation
SEM: Structural equation modeling
